# Physical and Chemical Properties of All-in-One Root Canal Irrigants: A Laboratory Study

**DOI:** 10.1055/s-0045-1812107

**Published:** 2025-10-22

**Authors:** Sonia Gupta, Rajkumar Narkedamalli, Krishna Prasad Shetty, Nidambur Vasudev Ballal

**Affiliations:** 1 Department of Conservative Dentistry and Endodontics, Manipal College of Dental Sciences, Manipal Academy of Higher Education, Manipal, India; 2 Department of Clinical Science, College of Dentistry, Centre of Medical and Bio-allied Health Science Research, Ajman University, Al-Jruf Ajman, United Arab Emirates

**Keywords:** demineralization, dual rinse HEDP, root canal irrigation, sodium hypochlorite, surface roughness, Triton, tissue dissolution

## Abstract

**Objective:**

This *ex vivo* study aimed to compare the physical and chemical properties of two all-in-one root canal irrigants—Dual Rinse HEDP (DR HEDP) and Triton—with respect to their soft tissue dissolution efficacy, effect on dentin surface roughness, and demineralization potential.

**Materials and Methods:**

Forty extracted human single-rooted teeth were used to evaluate soft tissue dissolution and demineralization, while 40 dentin slices from 20 teeth were used for surface roughness analysis. Irrigants tested included DR HEDP, Triton, NaOCl (positive control) alone, and distilled water (negative control). Soft tissue dissolution was assessed using standardized pulp tissue analogs. Calcium ion release was measured via atomic absorption spectrometry to assess demineralization. Surface roughness was analyzed using atomic force microscopy. Statistical analysis included analysis of variance and Tukey's post hoc tests with significance set at *p* < 0.05.

**Results:**

DR HEDP and NaOCl alone showed the highest tissue dissolution (36.49 and 35.57%, respectively, *p* > 0.05), significantly outperforming Triton (29.50%, *p* < 0.001). Triton exhibited the greatest dentin surface roughness (191.37 nm), significantly higher than DR HEDP (104.25 nm) and NaOCl (101.24 nm) ( *p* < 0.001). Calcium ion release was highest in the Triton group (135.61 mg/L), followed by DR HEDP (49.66 mg/L) and NaOCl (26.06 mg/L), with all intergroup differences being significant ( *p* < 0.001), except between DR HEDP and NaOCl ( *p* > 0.05).

**Conclusion:**

DR HEDP exhibited superior soft tissue dissolution similar to NaOCl, while causing significantly less dentin surface roughness and demineralization than Triton.

## Introduction

The successful outcome of root canal therapy is determined by the methodology and efficacy of instrumentation, irrigation, disinfection, and three-dimensional (3D) obturation of the entirety of the root canal system. 1 The intricate structure of the root canal system is of prime importance in this regard. Complex microanatomical features, including isthmuses, lateral canals, and apical ramifications, can provide shelter for biofilms and soft tissue debris. These remnants may subsequently lead to reinfection and act as a nutrient source for microbes in root canals that have undergone previous treatment. 2 As a result, clinicians widely accept that the more intricate the endodontic anatomy, the greater is the necessity for enhanced methods of chemo-mechanical root canal debridement. This process heavily depends on the application of irrigation solutions. Various agents have been recommended for endodontic irrigation. Among these, sodium hypochlorite (NaOCl) is regarded as the gold standard and a premier choice, 3 as it possesses both tissue dissolution and antimicrobial properties. 4 5 The pulpal tissue dissolution is a highly desirable characteristic of any irrigating solution, as it facilitates both the cleansing of the root canal system and the removal of necrotic tissue.

Mechanical instrumentation of root canals results in the formation of smear layer on the walls of the canal. This layer comprises inorganic dentin particles and organic matter, including pulp tissue, odontoblastic processes, necrotic material, microorganisms, and their metabolic byproducts. 6 These components can accumulate in the intricate structures of the root canal system, such as fins and ramifications. 7 8 Consequently, along with NaOCl, the use of an additional decalcifying substance is advised. In the past, this was accomplished by switching between the NaOCl irrigant and a complementary solution containing ethylenediaminetetraacetic acid (EDTA). 9 However, EDTA presents with certain limitations such as its decreased efficacy in removal of smear layer from apical third of the root canal system 10 and its cytotoxicity. 11

Furthermore, it substantially reduces the calcium-phosphate ratio of root dentin, which subsequently alters the permeability, surface roughness, and microhardness of the root dentin. 12

Of late, a new dental product containing 1-hydroxyethylidene-1,1-diphosphonic acid (HEDP), called the Dual Rinse HEDP (DR HEDP), has been introduced to the market by Medcem GmbH in Weinfelden, Switzerland. This product, supplied in a capsule form, comprises of 0.9 g of etidronate powder, which is to be mixed with 10 mL of NaOCl solution (0.5–5.25%) just before use in order to create an all-in-one irrigant. 13 This formulation allows dental professionals to employ DR HEDP solution in a “continuous chelation” method for root canals in the instrumentation phase and through the entire duration of treatment session. 14 Primary benefit of DR HEDP is its ability to function as a single irrigant for both cleaning and disinfecting root canals, significantly reducing irrigation time. 15

According to Rath et al, 16 employing a continuous chelation protocol led to a more uniform organic and inorganic composition of the dentin surface compared with sequential chelation using NaOCl followed by EDTA. Earlier research has shown that DR HEDP exhibits antimicrobial properties similar to NaOCl while being nontoxic. 17 18 Additionally, DR HEDP matches NaOCl in its ability of organic tissue dissolution 19 and enhances the adhesion of sealers and cements to root canal dentin. 20 21 22

A new all-in-one endodontic irrigation solution called Triton has been introduced by Brasseler in Savannah, United States. This product is composed of various chemical compounds, including 1,2,4-butanetricarboxylic acid, 2-phosphono, citric acid, sodium dodecylbenzenesulfonate, alcohol, polyethylene glycol 4-(tert-octylphenyl) ether, sodium lauryl sulfate, 2-ethylhexyl sodium sulfate, sodium cumenesulphonate, sodium hydroxide, and sodium hypochlorite. The solution comes in two separate components, part A and part B, which are combined by employing an automix technique to create a single solution prior to application.

Current research has shown that Triton outperforms traditional irrigants such as NaOCl and EDTA in removing debris and smear layers, 23 as well as dissolving soft tissues. 24 A recent study demonstrated Triton to have enhanced antimicrobial activity and organic tissue dissolution efficacy when compared with EndoJuice. 25

Till date, literature presents with no studies that have compared the physical and chemical properties of DR HEDP and Triton. Hence, the objectives of the present *ex vivo* study were (1) to evaluate the efficacy of DR HEDP and Triton on soft tissue dissolution; (2) to evaluate the effect of DR HEDP and Triton on surface roughness of root canal dentin; and (3) to determine the demineralization effect of a DR HEDP and Triton on root canal dentin.

The null hypotheses tested in the study were: (1) there is no difference in the ability of DR HEDP and Triton in dissolving soft tissue; (2) there is no difference in the surface roughness produced by DR HEDP and Triton; and (3) there is no difference in the demineralization produced by DR HEDP and Triton.

## Materials and Methods

Ethical clearance for the use of human extracted teeth for the experiments performed in the present study was obtained from the institutional review board prior to the commencement of the study (555/2024).

### Sample Size Estimation

The G*Power software (Heinrich Heine University, Dusseldorf, Germany) was used to estimate sample size. With 95% confidence level, 90% power, standard deviation of 0.15, and mean difference of 0.217, the minimum sample size was calculated to be 10 for each group for all the parameters tested in this study. 19 26 27

### Soft Tissue Dissolution

#### Sample Preparation

Forty extracted human single-rooted teeth with fully formed apices containing one straight single were selected. All the teeth were radiographed and analyzed under magnifying loupes (EyeMag Smart, Carl Zeiss, Oberkochen, Germany) to verify the presence of a single canal with closed apex, and the absence of intraradicular resorption, caries, cracks, or root canal filling. Superficial soft tissues on the root surface were removed with a curette and the teeth were stored in saline with 0.2% sodium azide (Millipore Sigma, St. Louis, Missouri, United States) at 4°C until use. The teeth were decoronated using a diamond disc (Horico Dental, Berlin, Germany) to standardize the root length. Working length was established by inserting a size 10 K file (Dentsply Sirona Endodontics, Ballaigues, Switzerland) into each root canal until it was just visible at the apical foramen and by subtracting 1 mm from the recorded length. Chemo-mechanical preparation was performed using ProTaper Universal nickel-titanium rotary instruments (Dentsply Sirona), with a torque control electric motor (X-Smart). The root canals were instrumented to size F3 (30/6%). Irrigation was performed using 2 mL of a DR HEDP (Medcem) combined with 3% NaOCl solution (Vista Dental, Racine, Wisconsin, United States) for 1 minute between each instrument change. One capsule (0.9 g) of DR HEDP powder was mixed with 10 mL of 3% NaOCl solution. Canals were finally irrigated with 5 mL of distilled water for 1 minute. The canals were dried with sterile paper points (Dentsply Sirona). Horizontal reference grooves of 0.5 mm depth were first made on the buccal and lingual surfaces of middle third of each root to serve as a guide for indicating the standard locations where simulated resorptive cavities were to be made later in the root canal. Each root specimen was then embedded in Eppendorf tubes (Merck, Darmstadt, Germany) using a putty impression material (3M ESPE, St. Paul, Minnesota, United States). Once that material set, the roots were removed from the vials to enable assembly and reassembly of the specimens. Each root was then sectioned longitudinally into two halves using a diamond disc (Horico Dental) under water cooling. Semicircular cavities were then be made on the root canal in the middle third (at the previously marked reference groove) of each root half using no. 6 round diamond point (Horico Dental) with slow speed under water cooling. The bur was marked 2 mm from its tip using a permanent marker, to ensure precise depth of the cavity. The depth of resorptive defect was standardized to 4 mm, which was confirmed by a digital caliper. The resorptive cavities were then irrigated with 5 mL of DR HEDP (Medcem) combined with 3% NaOCl (Vista Dental) for 1 minute to remove the smear layer. Finally, the cavities were flushed with 5 mL of distilled water for 1 minute and dried using paper points (Dentsply Sirona) ( Fig. 1 ).

**Fig. 1:**
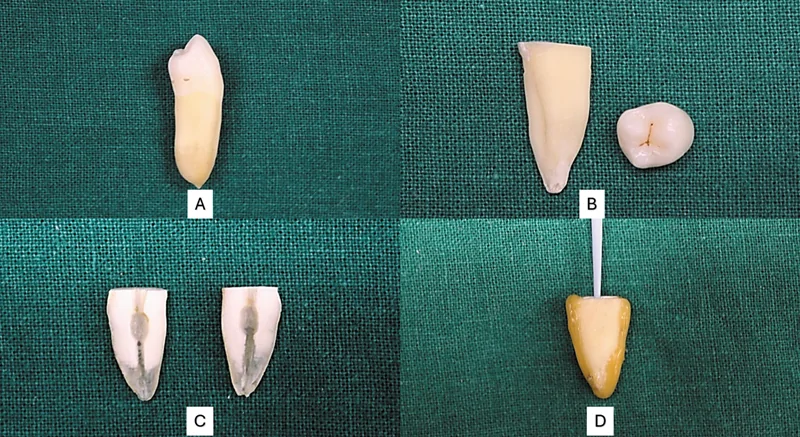
Schematics illustrating the stages of specimen preparation for the analysis of soft tissue dissolution: ( **A** ) intact single-rooted tooth; ( **B** ) decoronation; ( **C** ) simulated resorptive cavities; and ( **D** ) reassembled root halves maintaining canal patency.

#### Soft Tissue Preparation

Freshly extracted shrimp meat was employed for the experiment to simulate pulp tissue. The tissue samples used were standardized in weight and placed in the resorptive cavities of both halves of each root. Prior to weighing, each piece of tissue was blotted on Whatman filter paper (Merck, Darmstadt, Germany), dried, and then weighed using a precision balance placed in an airtight container (AT 261, Mettler, Greifensee, Switzerland). Both root halves were then reassembled using a light-curing resin barrier (OpalDam, Ultradent Products, South Jordan, Utah, United States). Patency was maintained by placing an F3 ProTaper gutta-percha point (Dentsply Sirona) between the canals of the two root sections. The apex of each root was sealed with sticky wax to simulate a closed-end system. A plastic tube of 3 mm height was glued at the cementoenamel junction (CEJ) of each root to act as a reservoir for the irrigating solution. Each root was then placed in its respective Eppendorf vial, and the samples were randomly divided using a sequence generator ( www.random.org ) into four groups ( *n* = 10) based on the irrigation regimen described below.

#### Irrigation Techniques

The irrigant volume was standardized to 5 mL, and the irrigation time was 5 minutes for all samples.

Group 1: 3% NaOCl solution (Vista Dental) combined with DR HEDP (Medcem)
Group 2: Triton (Brasseler)
Group 3: 3% NaOCl (Vista Dental)
Group 4: Distilled water (control)

In the DR HEDP group, one capsule of DR HEDP (0.9 g) was dissolved in 10 mL of the 3% of NaOCl (Vista Dental) solution and used immediately. In the Triton group, using the automix technique, the solutions from both components (A and B) were aspirated using a syringe as recommended by manufacturer and were used immediately. All irrigating solutions were introduced into the canal using a 30-gauge side-vented stainless steel needle (Vista Dental). The needle tip was inserted 1 mm short of the working length in each canal. After the irrigation protocols, the canals in groups 1, 2, and 3 were flushed with 5 mL distilled water for 1 minute to remove any precipitate that may have formed. The roots were then removed from the Eppendorf vials, split into two halves, and the meat tissue from the resorptive cavities was reweighed. Before weighing, all meat specimens were blotted dry on Whatman filter paper (Merck). All measurements were performed by a single blinded investigator who was unaware of the test solutions used. The percentage of tissue weight loss or gain was calculated by subtracting the posttreatment weight from the initial weight, dividing by the original weight, and multiplying by 100 ( Fig. 2 ).

**Fig. 2:**
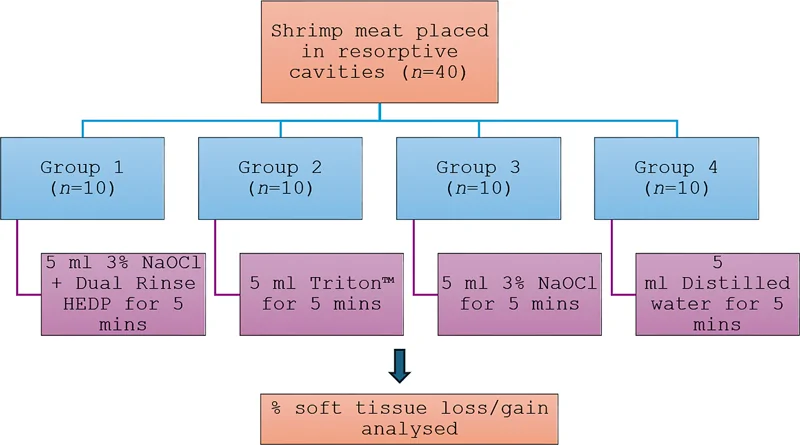
Flowchart of the methodology employed for soft tissue dissolution.

## Demineralization Analysis

### Sample Preparation

Forty intact human single-rooted permanent mandibular first premolars, with fully formed apices and single straight canals were obtained from individuals aged between 18 and 30 years. The teeth were extracted for orthodontic reasons. The rationale for selecting a relatively young age group (orthodontic patients) is to avoid the confounding variable of excessive calcification of root canal dentin. All the samples were radiographed and examined under magnifying loupes (EyeMag Smart) to verify canal anatomy and exclude teeth with resorption, calcification, caries, cracks, or previous endodontic treatment. Superficial soft tissue remnants on the root surfaces were removed using a curette, and the teeth were stored in saline with 0.2% sodium azide (Millipore Sigma) at 4°C until use.

#### Irrigation Techniques

Samples were randomly divided into four groups ( *n* = 10) using the aforementioned technique. Chemo-mechanical preparation was performed using ProTaper Universal NiTi rotary instruments (Dentsply Sirona) with an electric motor (X-Smart, Dentsply). Root canals were instrumented to F3 (30/0.06). Between each file, 2 mL of the experimental irrigant solution was delivered for 1 minute, followed by 5 mL of final rinse with the same irrigant solution. Irrigants were introduced using a 30-gauge side-vented needle (Vista Dental) inserted 1 mm short of the working length. The irrigant effluent from each sample was collected in a test tube placed beneath the Eppendorf tube holding the specimen. These aliquots were prepared for atomic absorption spectrometry (AAS) to quantify calcium ion concentration.

#### AAS Analysis

Calcium levels were determined using a flame atomic absorption spectrometer (iCE 3000 series, Thermo Fisher Scientific Inc., United States) at a wavelength of 422.7 nm. Due to high calcium concentrations in the test samples, a 10-fold dilution exceeded the upper detection limit of the instrument. Therefore, all experimental samples were diluted 100-fold prior to analysis to bring the concentrations within the linear range (0.05–3.0 mg/L) and simplify calculations. A sensitivity check was performed at 0.5 mg/L, with an expected absorbance range of 0.25 to 0.50 AU. Background correction was employed. Standard stock solutions (1000 µg/mL) were diluted to prepare working standards (1, 3, 5, and 10 µg/mL), and a calibration curve was constructed ( *R* ^2^ = 0.992) ( Fig. 3 ).

**Fig. 3:**
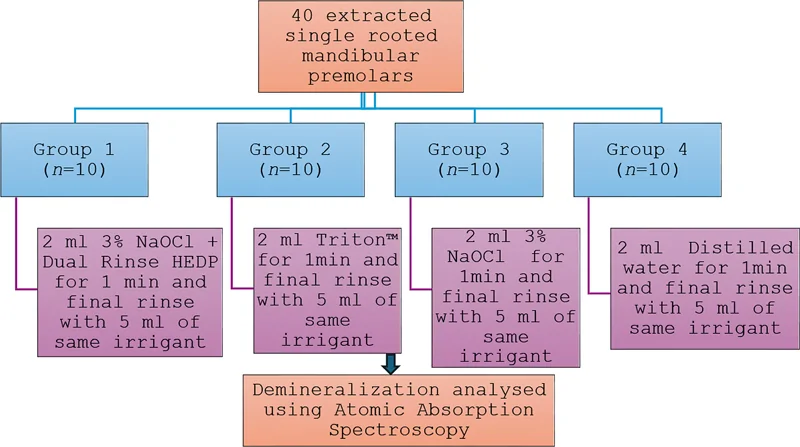
Flowchart of the methodology employed for demineralization analysis.

### Surface Roughness Analysis

#### Sample Preparation

Twenty extracted human single-rooted teeth were selected based on the same criteria as mentioned above for the demineralization analysis. Teeth were decoronated at the CEJ using a diamond bur (Horico Dental) under water coolant. Each root was longitudinally sectioned into two dentin slices using a diamond saw microtome (Leica SP 1600, Leica Microsystems, Wetzlar, Germany). Forty dentin segments were horizontally embedded in autopolymerizing acrylic resin (Meliodent RR, Kulzer, Hanau, Germany), exposing the root canal dentin surface. The dentin surface of each root segment was polished with a grinder-polisher, under water using a series of silicon carbide papers with increasing fineness (500, 800, 1,000, and 1,200 grit). Polishing was performed under distilled water. Final polishing was accomplished using 0.1 μm alumina suspension (Ultra-Sol R; Eminess Tec Inc, Monroe, North Carolina, United States) on a rotary felt disk. The samples were then randomly divided into four groups ( *n* = 10) using the aforementioned technique. Each sample was immersed in a glass plate containing 15 mL of the respective experimental solution for 6 minutes at room temperature. All the glass plates were placed on a vibrator to agitate the experimental solutions. The experimental solutions were renewed after the treatment of each specimen. Thereafter, the specimens were rinsed with 50 mL of distilled water and blotted dry.

#### Surface Roughness Analysis

Surface roughness (Ra, µm) was measured using atomic force microscopy (AFM; Veeco, Santa Barbara, California, United States) in contact mode with silicon nitride tips. The instrument was calibrated using polyethylene spheres. Scans were performed by a blinded, trained operator. Three regions (10 × 10 µm ^2^ ) per specimen were imaged, and the average Ra value was computed for each specimen using image analysis software ( Fig. 4 ).

**Fig. 4:**
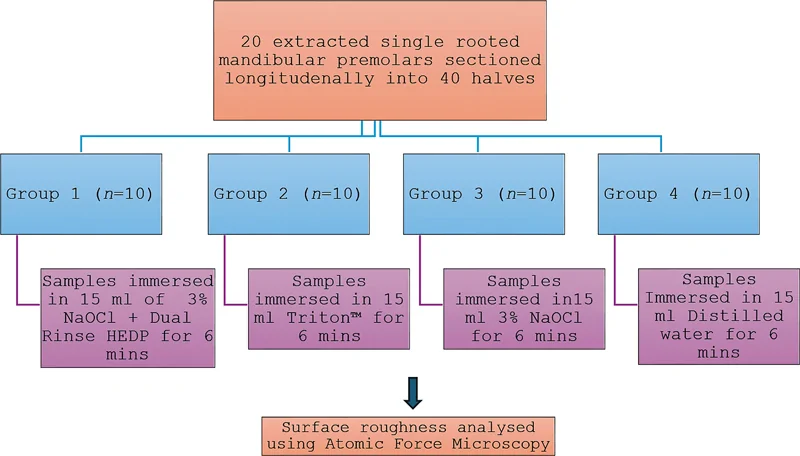
Flowchart of the methodology employed for surface roughness analysis.

### Statistical Analysis

Statistical analysis was performed using IBM SPSS Statistics version 26 (IBM Corp., Armonk, New York, United States). The normality of data for all three quantitative parameters (soft tissue dissolution, surface roughness, and demineralization) was assessed using the Kolmogorov–Smirnov and Shapiro–Wilk tests. One-way analysis of variance was applied to compare the mean values of the three parameters among the four experimental groups. Tukey's honest significant difference test was conducted for post hoc pairwise comparisons to identify intergroup differences. The significance level was set at *p* < 0.05. All values were reported as mean ± standard deviation, along with 95% confidence intervals.

## Results

### Soft Tissue Dissolution

Fig. 5 summarizes the soft tissue dissolution data for the control and experimental groups. Preirrigation tissue weights were not significantly different across the experimental and control groups ( *p* = 0.859). Postirrigation tissue weights varied significantly among the experimental groups. DR HEDP (36.49 ± 2.77%) and NaOCl alone (35.57 ± 1.80%) demonstrated the highest tissue dissolution with no significant difference between them ( *p* = 0.589). Triton (29.50 ± 1.37%) exhibited significantly less tissue dissolution compared with both DR HEDP and NaOCl alone groups ( *p* < 0.001). Distilled water, the negative control, showed no tissue dissolution effect (1.67 ± 0.15%).

**Fig. 5:**
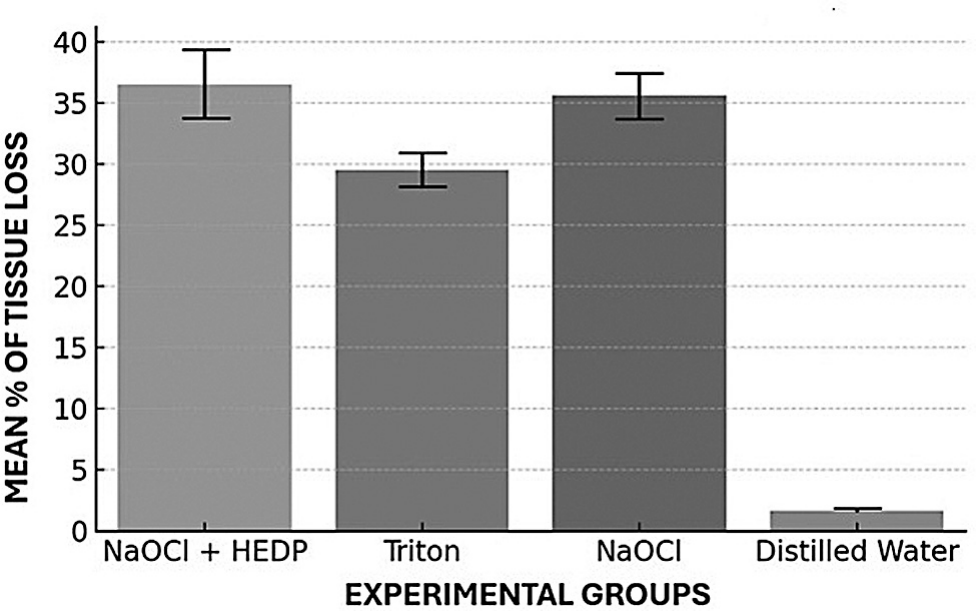
Mean percentage of soft tissue dissolution in different experimental groups. Bars represent the mean % of tissue loss observed with DR HEDP, Triton, NaOCl, and distilled water. Error bars indicate standard deviation.

#### Demineralization Effect

Fig. 6 summarizes the demineralization data for the control and experimental groups. Samples treated with Triton™ released the highest amount of calcium ions (135.61 ± 3.99 mg/L), compared with DR HEDP (49.66 ± 3.05 mg/L) and NaOCl alone (26.06 ± 3.18 mg/L) groups ( *p* < 0.001). There was no significant difference between DR HEDP and NaOCl alone groups ( *p* > 0.05). Samples treated with distilled water showed the least amount of calcium ion release (36.39 ± 2.96 mg/L).

**Fig. 6:**
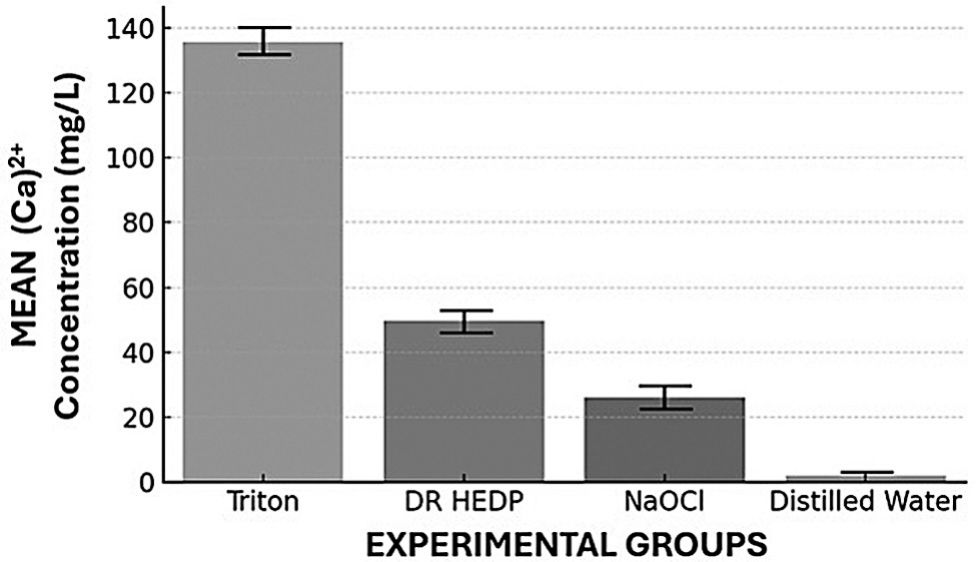
Mean calcium ion concentration (mg/L) in different experimental groups. Bars depict the mean (Ca ^2+^ ) concentration measured for Triton, DR HEDP, NaOCl, and distilled water. Error bars represent standard deviation.

#### Surface Roughness

Fig. 7 summarizes the surface roughness data for the control and experimental groups. Triton demonstrated the highest mean surface roughness (191.37 ± 3.95 nm), compared with the DR HEDP (104.25 ± 3.02 nm) and NaOCl alone (101.24 ± 4.06 nm) groups ( *p* < 0.001). There was no significant difference between the DR HEDP and NaOCl alone groups ( *p* = 0.244). Distilled water showed the least surface roughness (36.39 ± 2.96 nm). Fig. 8 demonstrates the 3D AFM images of dentin surfaces after treating with DR HEDP, Triton, NaOCl, and distilled water.

**Fig. 7:**
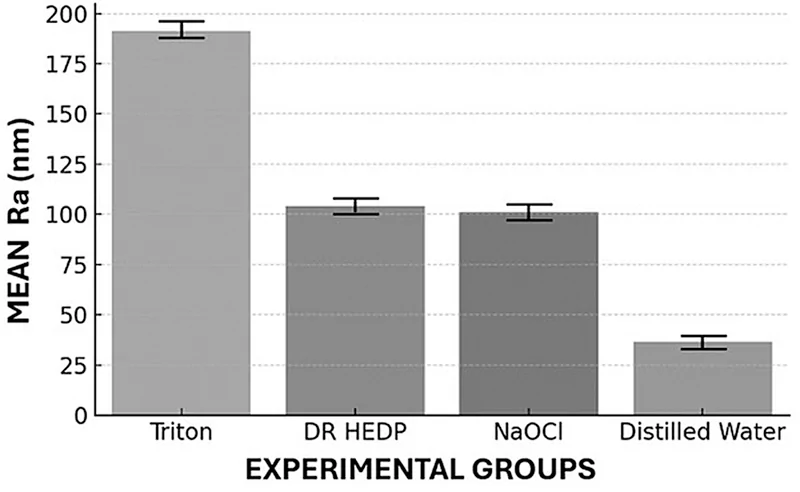
Mean surface roughness in different experimental groups. Bars represent the mean surface roughness observed with DR HEDP, Triton, NaOCl, and distilled water. Error bars indicate standard deviation.

**Fig. 8:**
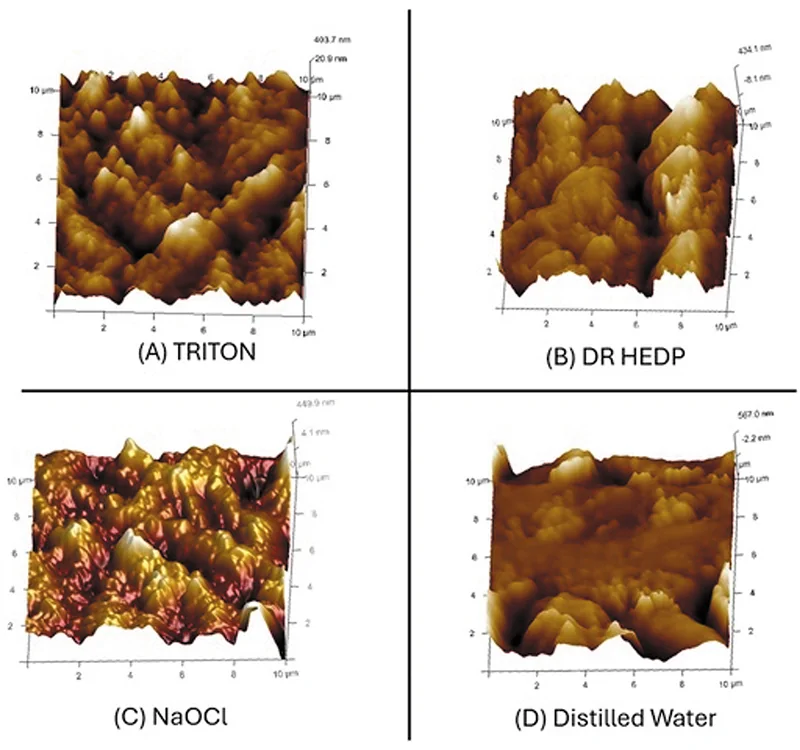
Three-dimensional atomic force microscopic images of root canal dentin surfaces after treatment with the experimental agents. Triton had maximum rough surface compared with DR HEDP and NaOCl. Distilled water showed minimal surface roughness. ( **A** ) Triton; ( **B** ) DR HEDP; ( **C** ) NaOCl; ( **D** ) distilled water.

## Discussion

Previous literature evaluated the individual performance of DR HEDP and Triton when they were used as root canal irrigants. 13 25 28 However, no comparative studies have evaluated the effectiveness of these two all-in-one irrigants in tissue dissolution, surface roughness, and their demineralizing potential. The findings of the present study demonstrated that Triton had less soft tissue dissolution ability, compared with NaOCl alone and the combination of NaOCl with HEDP (DR HEDP). Hence, the first null hypothesis that “there is no difference in the ability of DR HEDP and Triton in dissolving soft tissue” has to be rejected. This result supports the earlier work by Ballal et al, 17 who reported that etidronate does not interfere with NaOCl's proteolytic effect when used in continuous chelation. Novozhilova et al 29 observed that etidronate-containing irrigants maintained stable chlorine levels and dissolution efficacy over time, further validating the superior soft tissue dissolution efficacy of DR HEDP. The poor soft tissue dissolution performance of Triton could be attributed to its complex formulation, which includes acidic and surfactant components that could reduce the availability of active chlorine when premixed, thereby compromising its proteolytic activity. Deepa et al 24 reported that while Triton is an effective tissue dissolving agent, its activity under standard conditions may be less than that of traditional NaOCl-based irrigants.

When the surface roughness analysis of the irrigants was examined, samples treated with Triton demonstrated the highest surface roughness when compared with DR HEDP and NaOCl alone. Hence, the second null hypothesis that “there is no difference in the surface roughness produced by DR HEDP and Triton” must be rejected. The increased surface roughness of root dentin caused by Triton can be majorly attributed to the presence of citric acid and 1,2,4-butanetricarboxylic acid in its formulation. Citric acid, being a strong chelator, removes smear layer effectively and causes dentin erosion, which might have increased the surface roughness. 30 31 32 1,2,4-Butanetricarboxylic acid is a well-known chelating agent with both phosphonic acid and carboxylic acid groups, which would have enhanced the effect of Triton in smear layer removal, thus increasing the surface roughness. The category of surfactant present in Triton could also contribute to its superior smear layer removal leading to increased surface roughness. Incorporation of surfactants may improve the efficiency of an irrigant by decreasing surface tension and improving wettability, thereby enabling superior contact with the root canal walls. In contrast, DR HEDP exhibited a roughness profile comparable to NaOCl alone, indicating that etidronate exerts only a gentle chelating effect 28 and does not significantly exacerbate surface degradation. This aligns with previous research where DR HEDP was found to preserve the organic–inorganic balance of dentin and enhance adhesive outcomes without overetching. 16 20 Clinically, excessive roughness may compromise the structural integrity of root dentin and increase susceptibility to crack formation during obturation, 33 34 35 underscoring the importance of preserving dentin morphology during irrigation.

The decalcification effects of the experimental irrigants revealed that, samples treated with Triton had the most superior release of calcium ions, compared with both DR HEDP and NaOCl. Hence, the third null hypothesis that “there is no difference in the decalcification produced by DR HEDP and Triton” must be rejected. These findings are in accordance with Castagnola et al, 23 who observed marked smear layer removal and deep dentin demineralization following Triton irrigation. The higher demineralization potential of Triton could be owed to the acidic pH of citric acid present in its formulation. 36 37 Apart from this, the chelating activity of 1,2,4-butanetricarboxylic acid could have contributed to the increased demineralization potential of Triton. Conversely, DR HEDP released approximately half the amount of calcium as Triton, supporting its categorization as a soft chelator, due to its less aggressive action in terms of demineralization. 28 These results are consistent with the longstanding view that etidronate preserves dentin mineral content while still facilitating sufficient smear layer removal. 13 14 The NaOCl when used alone had minimal dentin decalcification effect due to its poor chelating efficacy. 38

Sodium hypochlorite is utilized as an endodontic irrigant with the concentrations ranging from 0.5 to 8.25%, without a universally accepted consensus on the optimum concentration. 33 39 The current study employed the use of 3% NaOCl as an irrigant since previous studies showed that more concentrated solutions could affect the mechanical properties of root canal dentin. 40 Apart from this, 3% NaOCl when mixed with HEDP stayed relatively stable for up to 2 hours, with minimal loss of free available chlorine. This presents optimum working time for clinicians by decreasing the need for chairside mixing. 13

The significance of the present work lies in its capacity to identify the more effective and less detrimental root canal irrigant that might prolong the survival of root canal-treated teeth. DR HEDP when used as an all-in-one irrigant provided optimal soft tissue dissolution, and minimal damage to root canal dentin matrix. In contrast, Triton appeared to be more aggressive toward dentin. This might potentially affect the structural and mechanical properties of root canal dentin, a significant consideration in the success of root canal treatment. By decreasing the structural damage to root dentin matrix, DR HEDP when used in a continuous chelation approach may potentially enhance the outcome of root canal therapy. Future research must focus on validating the presented findings in a clinical setting and explore the long-term follow-ups of these irrigation protocols.

The present work was an *ex vivo* preliminary investigation into the physical and chemical properties of Triton and DR HEDP. Results of this study present with a few limitations that may not necessarily be generalized in clinical situations. First, the soft tissue dissolution model employed shrimp meat, which is although analogous to human pulp tissue, it lacks the vital responses and vasculature of *in situ* pulp tissue. Also, this study also employed single-rooted human teeth with standardized cavities in the root canals, which may not have provided an overall representation of the varied root canal anatomies encountered in day-to-day clinical situations. Second, the evaluation of dentin surface roughness and demineralization was performed on standardized root slices, which may not capture the regional variations observed in clinical root canals.

Considering the limitations of the current *ex vivo* study, it may be concluded that DR HEDP and NaOCl alone produced minimal dentin surface roughness and demineralization, with superior soft tissue dissolution efficacy compared with Triton.
